# Physical activity, sedentary behavior and their correlates in children with Autism Spectrum Disorder: A systematic review

**DOI:** 10.1371/journal.pone.0172482

**Published:** 2017-02-28

**Authors:** Rachel A. Jones, Katherine Downing, Nicole J. Rinehart, Lisa M. Barnett, Tamara May, Jane A. McGillivray, Nicole V. Papadopoulos, Helen Skouteris, Anna Timperio, Trina Hinkley

**Affiliations:** 1 Deakin University, Institute for Physical Activity and Nutrition, School of Exercise and Nutrition Sciences, Geelong, Victoria, Australia; 2 Early Start Research Institute, Faculty of Social Sciences, University of Wollongong, Wollongong, New South Wales, Australia; 3 Deakin University, Deakin Child Study Centre, School of Psychology, Burwood, Victoria, Australia; 4 Deakin University, School of Health and Social Development, Burwood, Victoria, Australia; 5 University of Melbourne, Department of Paediatrics, Parkville, Victoria, Australia; 6 Murdoch Childrens Research Institute, Parkville, Victoria, Australia; 7 Deakin University, Centre of Social and Early Emotional Development, School of Psychology, Burwood, Victoria, Australia; Vanderbilt University, UNITED STATES

## Abstract

Autism Spectrum Disorder affects up to 2.5% of children and is associated with harmful health outcomes (e.g. obesity). Low levels of physical activity and high levels of sedentary behaviors may contribute to harmful health outcomes. To systematically review the prevalence and correlates of physical activity and sedentary behaviors in children with Autism Spectrum Disorder, electronic databases (PsycINFO, SPORTDiscus, EMBASE, Medline) were searched from inception to November 2015. The review was registered with PROSPERO (CRD42014013849). Peer-reviewed, English language studies were included. Two reviewers screened potentially relevant articles. Outcomes of interest were physical activity and sedentary behaviour levels and their potential correlates. Data were collected and analysed in 2015. Of 35 included studies, 15 reported physical activity prevalence, 10 reported physical activity correlates, 18 reported sedentary behavior prevalence, and 10 reported sedentary behavior correlates. Estimates of children’s physical activity (34–166 mins/day, average 86 mins/day) and sedentary behavior (126–558 mins/day in screen time, average 271 mins/day; 428–750 mins/day in total sedentary behavior, average 479 mins/day) varied across studies. Age was consistently inversely associated, and sex inconsistently associated with physical activity. Age and sex were inconsistently associated with sedentary behavior. Sample sizes were small. All but one of the studies were classified as having high risk of bias. Few correlates have been reported in sufficient studies to provide overall estimates of associations. Potential correlates in the physical environment remain largely unexamined. This review highlights varying levels of physical activity and sedentary behavior in children with Autism Spectrum Disorder. Research is needed to consistently identify the correlates of these behaviors. There is a critical need for interventions to support healthy levels of these behaviors.

## Introduction

Low levels of physical activity (PA) and high levels of sedentary behaviors (SB) are associated with short- and long-term health consequences from early childhood, through childhood and adolescence. Consequences include cardio-metabolic risk factors, impaired psychosocial well-being and cognitive functioning and poorer weight status [1–7]. However, across the world few children or adolescents (hereafter referred to as children) participate in at least 60 minutes per day of moderate- to vigorous-intensity physical activity (MVPA) as is recommended [8–13]. There are particular sub-groups whose risk of inactivity may be even higher, such as children (0–18 years) diagnosed with an Autism Spectrum Disorder (ASD).

ASD is clinically defined by impairments in social, communication and reciprocal interaction, with repetitive, restricted, and stereotypical behavioral patterns [14] and is now thought to affect up to 2.5% of children [15–17]. Social impairment and restricted interests combined with high rates of motor problems [18] commonly present in this disorder may limit the opportunities for engagement in PA and put children with ASD at risk of increased SB and other harmful health outcomes including and obesity [19]. Engaging in PA may offer opportunities for socialization with peers, broaden a child’s range of interests, increase motor skills and thus have positive impacts on a range of outcomes including physical health, social-emotional and developmental functioning [20]. Therefore, similar to typically developing (TD) children [21], children with ASD could potentially benefit from lifestyle modifications that promote increased PA and reduced SB. To date, there is no clear evidence documenting the prevalence of PA and SB in children with ASD, nor comprehensive evidence of the correlates of these behaviors. Understanding prevalence and correlates will inform future PA and SB interventions and identify potential strategies. Therefore, the aim of this review was to systematically examine reported prevalence of PA and SB, along with their correlates, in children with ASD.

## Methods

The guidelines outlined in the Preferred Reporting Items for Systematic Reviews and Meta-analyses (PRISMA) statement [22] were followed (S1 Table. Completed PRISMA checklist). The review was registered with PROSPERO (CRD42014013849); no protocol exists. A literature search was conducted using four databases: PsycINFO, SPORTDiscus with Full Text, EMBASE and Medline. Search strategies included key words in four categories: (1) population, (2) PA, (3) SB and (4) Autism Spectrum Disorder. Table 1 provides the full search strategy for PsycINFO, modified where necessary for the remaining databases. Searches were completed from inception of the databases to November 2015. Data were collected and analysed in 2015/2016.

**Table 1:** Search strategy used in PsycINFO.

1. Population	“school age*” or “young child*” or youth or adolesc* or teen* or preschool or early childhood or child* or infant or boy* or girl* or female* or male*
2. Physical activity	“physical* activ*” or exercis* or “motor activit*” or “locomotor activit*” or sport* or recreation*
3. Sedentary behavior	“sedentary behavio*” or sedentar* or “television view*” or "tv" or television or “video game*” or “screen time” or “electronic game*” or computer* or “small screen*” or e-game* or video* or "physical inactivity" or "screen based media" or gaming or “electronic media”
4. Autism spectrum disorder	Autis* or “Autism spectrum disorder” or ASD or Asperger’s* or “Pervasive Developmental Disorder” or “Autistic disorder” or “Pervasive Developmental Disorder Not Otherwise Specified” or PDD or PDDNOS
5. 2 or 3	
6. 1 and 4 and 5	
7. Limit 6 to peer-reviewed and English language and age groups: childhood (birth to 18 years)	

Papers were included if they were: (1) peer reviewed, written in English and available in full text; (2) included children aged 0–18 years with ASD; and (3) reported on the amount of time children spent in habitual PA/SB; or (4) reported a measure of association between PA/SB and another variable (e.g., sex). Case studies and studies that reported only qualitative data or measures of fitness and energy expenditure were excluded. Exclusion criteria were case studies and studies that reported fitness or aerobic fitness or energy expenditure. No sample characteristics, such as low or high functioning Autism, nor the presence of comorbidity, were considered as exclusion criteria to allow for the greatest number of papers to be considered for inclusion.

The following process was followed to ensure that all relevant papers were retrieved and included: (1) two authors initially screened titles (KD and RJ); (2) abstracts and full papers of those that were deemed to be relevant were then screened by the same two authors; and (3) data were extracted from included studies (KD). For steps one and two, where authors did not agree (<5%) resolution was achieved through discussion or a third author (TH) was consulted. For analyses focused on correlates, only one study reported results of multiple variable analyses with all studies reporting bivariable and unadjusted models. Therefore, results of unadjusted models are reported. Notations are made in tables where differences in unadjusted and adjusted models exist.

Prevalence of PA included average daily minutes of MVPA, total PA (counts per minute, CPM) or number of days being active. Where available, the percentage of children meeting the 60min MVPA international recommendations [23] was extracted. For SB, prevalence was reported as average daily minutes of screen time (TV watching, video gaming, etc.) or total sedentary time. Reported associations between behaviors and potential correlates were coded as positive (+), negative (-) or null (0). The number of studies supporting each association is given as a percentage of the total studies for that variable. These codes were then analyzed and given a summary code based upon the percentage of studies and direction of association as follows: 0–33% of studies reporting an association in a given direction were coded as 0 (no association); 34–59% of studies supporting an association in a given direction were coded as? (inconsistent association); and 60–100% of studies supporting an association in a given direction were coded as + or–(positive/negative association). Variables investigated four or more times were coded ++ (strong positive association),—(strong negative association), or 00 (strong null association) [24–26]. Only the variables that are reported four or more time are discussed. If variables are reported fewer than four times, the evidence was considered insufficient to make an assumption about the direction of association. Correlates were divided into subgroups: child, familial and environmental variables, aligning with the Ecological Model [27]. A meta-analysis was not completed due to too few studies investigating the same correlate and outcome.

Risk of bias was determined using a published six-component rating scale [28] and was assessed on: selection bias (e.g., representativeness), study design (e.g., longitudinal), confounders (e.g., education), blinding (e.g., outcome assessor aware of group allocation), data collection methods (validity, reliability), and withdrawals/dropouts (e.g., were they reported, percent of complete data: weak <60%; strong ≥80%). A risk of bias score for each subsection was calculated as per the standardized protocol (1 = low risk, 2 = moderate risk, 3 = high risk). If a component was not described it was rated as high risk of bias. Once individual components were assessed, a global score for each paper was calculated (1 = low risk [3 low risk ratings, no high risk ratings], 2 = moderate risk [<3 low risk ratings, 1 high risk rating], 3 = high risk [>1 high risk rating]). The data extraction tables are provided in the supporting information (S2 Table. Data extraction table for the physical activity papers; S3 Table. Data extraction tables for the sedentary behavior papers)

## Results

**Fig 1:**
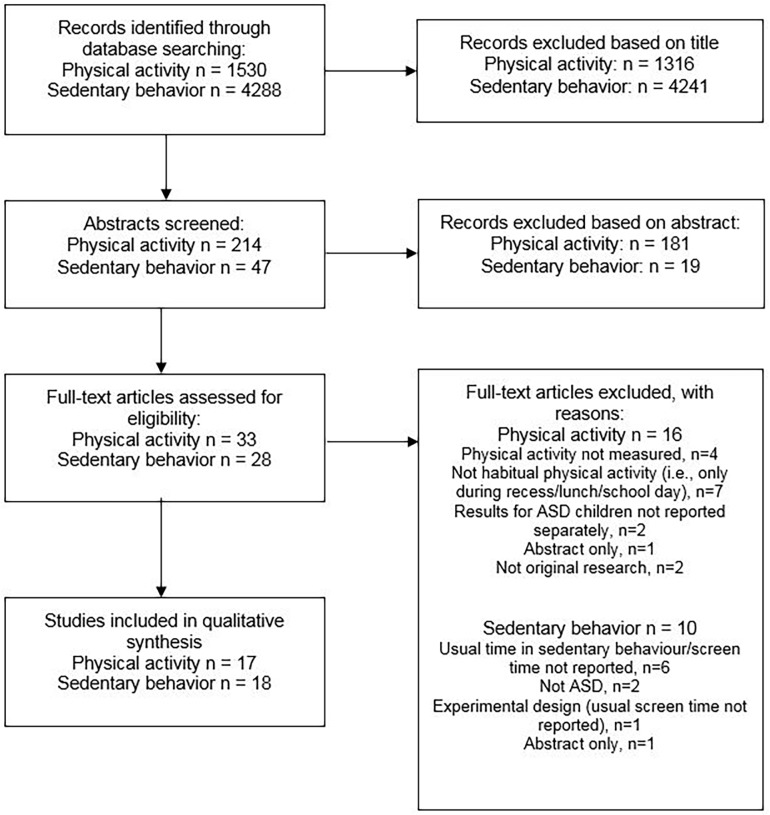
Flow diagram of search results.

Thirty-five studies met the inclusion criteria: 15 studies reported PA prevalence [29–43]; 10 reported PA correlates [29, 30, 32–35, 37, 42, 44, 45]; 18 reported SB prevalence [32, 37–39, 43, 46–57]; and 10 reported SB correlates [32, 37, 44, 47, 48, 50–52, 55, 56] (see Fig 1). Of these, five papers reported both PA and SB data [32, 37–39, 44].

For PA, four studies reported on preschool-aged children (2–5 years), 13 reported on primary school-aged children (5–12 years), 11 reported on secondary school-aged children (12–17 years) and two included participants aged 18–21 years. For SB, three studies included preschool-aged children; 12 included primary school-aged children; 14 included secondary school-aged children and two included adults. Studies typically included participants in more than one age range: the studies with adult participants were included as they also reported on participants aged 12–18 years but did not report data separately; therefore, all data from these studies are presented.

### Physical activity—Study characteristics

Table 2 highlights study characteristics of all 17 PA studies. Twelve were from the United States (70%), with the remainder from Iran [30, 45], Taiwan [35], Japan [40] and the UK [43]. Sixteen studies were cross-sectional [29–31, 33–45]. Six studies (35%) had fewer than 20 participants with ASD [31, 36, 38, 41–43], four (24%) involved 21–50 participants [33–35, 40], five (29%) involved 51–100 participants [29, 30, 37, 45, 52] and two (12%) had sample sizes of over 100 participants [32, 39]. One study had a sample size of 900 [39]; with that study removed as an outlier, the average sample size was 41 participants.

**Table 2:** Studies reporting habitual physical activity prevalence and/or correlates of physical activity.

Author, date, location	Sample^a^	ASD diagnosis	IQ	Instrument/ inclusion criteria	Average (SD) min/day MVPA	Average (SD) CPM	Risk of bias global score^b^
**Objectively measured physical activity**							
Ayvazoglu et al. 2015, USA	ASD: N = 6, ave age 7.5±3.4yrs (range 4-13yrs), 66% boys	Prior diagnosis	All without ID	Actigraph, 1000 vector magnitude used to identify 1 min interval of MVPA; Inclusion criteria not reported	**ASD**: 34 (SD not reported)	-	3
Boddy et al. 2015, UK	ASD: N = 9, sample characteristics not reported separately for children with ASD	Parent reported diagnosis	All with ID	Actigraph, MVPA ≥2296 CPM; ≥9h/day on weekday, ≥8h/day on weekend, ≥3 days	**ASD**: 48 (SE = 4)	-	3
Bandini et al. 2013, USA	ASD: N = 53, ave age 6.6±2.1yrs (range 3-11yrs), 83% boys; TD: N = 58, ave age 6.7±2.4yrs (range 3-11yrs), 78% boys	Study confirmed diagnosis (met ADI-R cutoff)	Mean IQ^c^ = 85.8, SD = 22.1	Actigraph, MVPA ≥1500 CPM; >600 m/day on 3 weekdays and 1 weekend day	**ASD**: 50; **TD**: 57 (not sig; p value not reported; SD not reported)	-	3
MacDonald et al, 2011, USA	N = 72, range 9-18yrs, 76% boys	Study confirmed diagnosis (met Social Responsiveness Scale cutoff)	9-11yrs group: Mean IQ^d^ = 82.92, SD = 19.69; 12-18yrs group: Mean IQ^d^ = 79.88, SD = 20.45	Actical, MVPA ≥1504 CPM; ≥10h/day on ≥3 weekdays and ≥1 weekend day	**ASD**: *9-11yrs group*: 132 (84); *12-18yrs group*: 90 (98)	-	3
Memari et al. 2012, Iran	ASD: N = 80, ave age 9.6±1.8yrs (range 7-14yrs), 69% boy	Prior diagnosis using DSM-IV-TR.	All with ID	Actigraph, cutpoints not reported; ≥8h/day on ≥3 weekdays and ≥1 weekend day	-	**ASD**:*7-18yrs*: 1763 (576), *9-10yrs*: 1657 (580), *11-12yrs*: 1265 (431), *13-14yrs*: 1146 (445)	2
Obrusnikova et al. 2011, USA	ASD: N = 14, ave age 10.6±1.7 (range 8-14yrs), 86% boys	Prior diagnosis, N = 10 Asperger Syndrome; N = 3 PDD-NOS; N = 1 Autism.	Not reported	Actical, MVPA ≥3 METs; calculated using a regression model; ≥10h/day (no. days not provided)	**ASD**: 82 (62)	-	3
Pan et al. 2005, USA	ASD: N = 30, ave age 13.2yrs, 90% boys	Parent reported diagnosis, N = 14 autism, N = 12 Asperger’s syndrome, N = 4 PDD-NOS	All without ID	Actigraph, MVPA ranged from ≥1017 (10yrs) to ≥2274 CPM (18yrs); ≥8h/day on ≥3 weekdays and ≥1 weekend day	**ASD**: 78 (SD not reported)	**ASD**: 490 (SD not reported)	3
Pan et al. 2006, USA	ASD: N = 30, ave age 13.2±2.1yrs (range 10-19yrs), stratified by elementary (ave age 10.7yrs), middle (ave age 13.3yrs), and high school (ave age 15.1yrs), 90% boys	Parent reported diagnosis, N = 14 autism, N = 12 Asperger’s syndrome, N = 4 PDD-NOS	All without ID	Actigraph, MVPA ranged from ≥1017 (10yrs) to ≥2274 CPM (18yrs); ≥8h/day on ≥3 weekdays and ≥1 weekend day	**ASD**: *Elementary*: 133 (79), *Middle*: 75 (33), *High*: 40 (19)	**ASD**: *Elementary*: 670 (374), *Middle*: 483 (128), *High*: 361 (120)	3
Pan et al. 2011, Taiwan	ASD: N = 35, ave age 9.6±0.5yrs (range 7–12 yrs), stratified by lower (ave age 7.6yrs), middle (ave age 9.6yrs), and upper grade (ave age 11.8yrs), 100% boys	Prior diagnosis using DSM-IV-TR; N = 14 Asperger’s syndrome, N = 22 mild autistic disorder	Not reported	Actigraph, MVPA ranged from ≥1017 (10yrs) to ≥2274 CPM (18yrs); 4 weekdays and 1 weekend day	**ASD**: Weekdays: *Lower grade*: 129 (20), *Middle grade*: 92 (18), *Upper grade*: 91 (16); Weekend days: *Lower grade*: 135 (50), *Middle grade*: 106 (39), *Upper grade*: 57 (26)	-	3
Sandt et al. 2005, USA	ASD: N = 15, ave age 9.5±1.9yrs (range 5-12yrs), 67% boys; TD: N = 13, ave age 8.9±2.0yrs (range 5-12yrs), 32% boys	No report on who performed diagnosis. N = 9 with autism, N = 2 Asperger syndrome, N = 4 with PDD-NOS	Not reported	Actigraph, cut points not reported; 4 weekdays and 1 weekend day	**ASD**: 128 (72), **TD**: 132 (46) (not sig; p value not reported)-		3
Tatsumi et al. 2015, Japan	ASD: N = 31, ave age 5.0yrs, 81% boys; TD: N = 16, ave age 5.5yrs, 63% boys	Prior diagnosis using DSM-IV	Not reported	Actiwatch, cut point not reported; Inclusion criteria not reported	-	Median values (range): Weekdays: *Morning (9am-12pm)*: **ASD**: 758 (687–937), **TD**: 803 (625–963); *Afternoon (12pm-6pm)*: **ASD**: 649 (511–694), **TD**: 740 (396–879); *Evening (6pm-sleep)*: **ASD**: 542 (423–603), **TD**: 578 (487–638); Weekend days: *Morning*: **ASD**: 631 (498–757), **TD**: 746 (708–930); *Afternoon*: **ASD**: 628 (506–797), **TD**: 790 (670–994); *Evening*: **ASD**: 517 (401–666), **TD**: 590 (448–738), (sig diff between ASD and TD children only during weekend mornings: p = 0.023)	3
Tyler et al. 2014, USA	ASD: N = 17, ave age 12.6±2.3yrs (range 5-12yrs), 53% boys; TD: N = 12, ave age 9.0±1.8yrs (range 5-12yrs), 50% boys	Study confirmed diagnosis (met ADOS cut off)	Verbal IQ mean = 60.1, SD = 25, range 16–110; Nonverbal IQ mean = 65.7, SD = 40, range 16–172	Actigraph, ≥500 CPM; Inclusion criteria not reported	**ASD**: 166 (59), **TD**: 218 (66) (p = 0.01)	-	3
Wachnob et al. 2015, USA	N = 10, ave age 11.8±2.3yrs (range 9-16yrs), 90% boys	Parent reported diagnosis	Not reported	Actigraph, cut point not reported; ≥8 hr/day	**ASD**: *Weekday*: 76 (92); *Weekend day*: 63 (63)	-	3
**Subjectively measured physical activity**							
Dreyer Gillette et al. 2015, USA	ASD: N = 900, range 10-17yrs, 84% boys; TD: N = 45000, range 10-17yrs, 50% boys	Parent reported diagnosis	Not reported	Parent report questionnaire, physical activity per day	% of sample physically active (i.e., engaged in exercise, played sport, or participated in physical activity for ≥20 mins that made them sweat and breathe hard) on the number of days specified: **ASD**: 18% 0 days, 30% - 1–2 days, 21% 4–6 days, 20% 7 days; **TD**: 10% 0 days, 27% - 1–2 days, 40% 4–6 days, 23% 7 days; (sig difference between % of ASD and TD children participating in 0 days and 4–6 days of physical activity only; p<0.05)		3
Memari et al. 2015,	N = 83, ave age 9.8±1.8yrs (range 6-15yrs), 64% boys	Study confirmed diagnosis (met ADI-R cutoff)	All with IQ>70	Goldin Shaphard Leisure Time Questionnaire	Not reported		3
Must et al. 2015, USA	ASD: N = 53, ave age 6.6±2.1yrs, 83% boys; TD: N = 58, ave age 6.7±2.4yrs, 78% boys	Study confirmed diagnosis (met ADI-R cutoff)	Not reported	Parent report organized and unstructured physical activity	Not reported		3
Orsmond et al. 2011, USA	N = 103, ave age 17.8±2.2yrs (range 12-21yrs), 76% boys, cohort	Study confirmed diagnosis (met ADI-R cutoff)	56% with ID	Parent report time use diary	**ASD**: 34 minutes of physical activity/day (SD not reported; range 0–287 min/day)		3

^a^ Cross sectional in design unless otherwise stated;

^b^ 1 = strong quality/low risk of bias, 2 = moderate quality/risk of bias, 3 = weak quality/high risk of bias;

^c^ measured using DAS General Conceptual Ability Score;

^d^ measured using 2 subtests from the WASI.

Abbreviations: ADI-R—revised Autism Diagnostic Interview; ADOS—Autism Diagnostic Observation Schedule; ave age—average age; ASD—children with Autism Spectrum disorder; CPM—counts per minute (total physical activity); DSM-IV-TR—Diagnostic and Statistical Manual of Mental Disorders; hr—hours; ID—intellectual disability; IQ—intelligence quotient; min—minutes; MVPA—moderate- to vigorous-intensity physical activity; PDD-NOS—pervasive developmental disorder not otherwise specified; SD—standard deviation; sig—significant; TD—typically developing children; yrs—years

Six studies reported a confirmed ASD diagnosis [29, 32, 37, 38, 44, 45], five used prior report of diagnosis [30, 31, 35, 40, 41], five used parent report [33, 34, 39, 42, 43], and one did not report how ASD diagnosis was made [36]. Three studies reported diagnosis using DSM-IV criteria [30, 33, 40]. Two studies only included participants with an intellectual disability (ID) [30, 43], three included participants without an ID [33, 34, 41], and one included participants with and without ID [32]. Four studies reported mean IQ, ranging from 60 to 86 [29, 37, 38, 45], and seven studies did not report the presence of ID or IQ [31, 35, 36, 39, 40, 42, 44].

Sixteen of the 17 PA studies were classified as having high risk of bias; only Memari et al [30] had moderate risk of bias. The only risk of bias subsection that consistently received a low rating was the data analyses procedures (S4 Table).

### Physical activity—Prevalence

Thirteen studies used objective measures of PA: Actigraph [29, 30, 33–36, 38, 41–43], Actical [31, 37], and Actiwatch [40] accelerometers with the remainder using parent-proxy report of PA [32, 39, 45, 52]. Eleven studies using accelerometers reported average minutes of MVPA per day (mean 90 minutes, range 34–165) [29, 31, 33–38, 41–43] and five reported CPM (mean 826 cpm, range 361–1763) [30, 33, 34, 36, 40]. Two studies reported both MVPA and CPM [33, 34]. Two studies collected PA prevalence data using a parent proxy report questionnaire [32, 39]. Data reported from these questionnaires were inconsistent: one reported hours of physical activity per day [32], and the other reported the percent of days participants were physically active [39].

Five studies reported PA prevalence for samples of ASD and typically developing (TD) children [29, 36, 38–40]; two studies [29, 38] did not report statistical assessment of difference between those samples for accelerometry data. However, Bandini et al. [29] reported both accelerometry and parent-report PA data. They found that there was no association between the two for either children with ASD or TD children but that TD children participated in significantly more types of activities, and more hours per year of activities, than children with ASD when measured by parent-report. Dreyer-Gillette [39] found that a significantly higher percent of children with ASD (18.8%) spent no days being physically active (20 mins or more in vigorous PA) compared with their TD counterparts (10.2%). Similarly, a significantly lower percent of children with ASD (31.3%) spent 4–6 days per week being physically active compared with TD children (39.8%). However, there were no differences in the percent of ASD and TD children who spent 1–3 or 7 days being active [39]. Tatsumi [40] reported that children with ASD had significantly lower CPM (630.5) on weekend day mornings compared with TD children (746.0); CPM did not vary for other times of the week or other periods of weekend days (afternoon, evening). The final study [36] reported no statistical difference in CPM between ASD and TD children. Seven studies reported prevalence of compliance with the 60 minutes of MVPA recommendation [29, 31, 33, 34, 36, 37, 43]: these varied between 21% and 100% for children with ASD [29, 31, 33, 34, 36, 37, 43] and 43% and 92% for TD children [29, 36].

### Physical activity—Correlates

Table 3 presents a summary of the potential correlates of PA reported in the studies. Across the 10 studies that reported correlates of PA, 24 potential correlates were identified. The most commonly studied correlates were child and familial variables. Only one environmental variable was reported. Of the potential correlates that were studied four or more times, age showed a consistent negative association and sex showed an inconsistent association with PA. Familial variables were not reported in a sufficient number of studies to determine an overall association. The only environmental variable investigated, day of the week (week vs. weekend day), was reported in four studies: overall findings suggest no differences in PA between week and weekend days.

**Table 3:** Correlates of physical activity among children diagnosed with Autism Spectrum Disorder.

Correlate	Associated with children’s PA (reference)	Association (+/-)	Not associated with children’s PA (reference)	Summary coding for row (n/N for row, %)	Summary code for association (-/+)
**Child Variables**					
Age	Memari [30], Pan [33], MacDonald [37]	-	Orsmond [32]	3/4 (75)	—
Sex	Memari [30] (boys only), Memari [45] (boys only)	+	Orsmond [32] MacDonald [37]	2/4 (50)	??
BMI z-score			Bandini [29]	0/1 (0)	0
School level/grade	Pan[34], Pan[35]	-		2/2 (100)	-
Sleep quality			Wachob [42]	0/1 (0)	0
Sedentary pursuits	Memari [30] (boys only), Pan [33] (boys and girls)	-		2/2 (100)	-
Comorbidities	Memari [30]	-		1/1 (100)	-
IQ			MacDonald [37]	0/1 (0)	0
ASD symptom severity			MacDonald [37] Memari [45]	0/2 (0)	0
Psychotropic medication (taking)			Memari [30]	0/1 (0)	0
**Familial variables**					
Parental education			Memari [45]	0/1 (0)	0
Maternal education	Orsmond [32]	+		1/1 (100)	+
SES			Memari [30]	0/1 (0)	0
Poverty income ratio	Memari [45]	+		1/1 (100)	+
Household structure	Memari [30] (single parent), Memari [45] (single parent)	-		2/2 (100)	-
Maternal marital status			Orsmond [32]	0/1 (0)	0
Family size			Orsmond [32]	0/1 (0)	0
Maternal MVPA			Pan [33]	0/1 (0)	0
Paternal MVPA			Pan [33]	0/1 (0)	0
Maternal CPM	Pan [33]	-		1/1 (100)	-
Paternal CPM	Pan [33]^a^			1/1 (0)	+
Maternal/paternal support for physical activity			Pan [33] (MVPA and CPM)	0/1 (0)	0
Perceived parental barriers	Must [44]	-		1/1 (100)	-
**Environmental**					
Day of week (week vs. weekend day)			Memari [30], Pan [34], Pan [21] Orsmond [32]	0/4 (0)	0

Abbreviations: CPM—counts per minute; LPA—light-intensity physical activity, MPA—moderate-intensity physical activity, MVPA—moderate- to vigorous-intensity physical activity; SES—socio-economic status.

Note:

^a^ in multivariable analyses, this association was attenuated and Paternal CPM was not associated with children’s PA.

### Sedentary behavior—Study characteristics

Table 4 shows the characteristics of included SB studies. Of the 18 studies that reported SB outcomes, 13 were from the United States (68%), three from Canada [55–57], one from Thailand [46] and one from the United Kingdom [43]. One study (6%) had fewer than 20 participants with ASD [43], four (22%) had between 21 and 50 participants [38, 47, 54, 56], seven studies (39%) had between 51 and 100 participants [37, 44, 46, 49, 52, 53, 57], and six studies had more than 100 participants [32, 39, 48, 50, 51, 55]. One study had a sample size of 900 [39]; with that study removed as an outlier, the average sample size was 82 participants. Most studies were cross sectional (16/18, 89%) [37–39, 43, 44, 46–55, 57] and used subjective measures of SB (e.g. parent report, 15/18, 83%) [39, 44, 46–55, 57]. Three studies reported objective measures of SB: one used Actical [37] and two used Actigraph [38, 43] accelerometers.

**Table 4:** Studies reporting habitual sedentary behavior prevalence and/or correlates.

Author, date, location	Sample^a^	ASD diagnosis	IQ	Instrument/ inclusion criteria	Measure	Average (SD) min/day	Risk of bias global score^b^
**Objectively measured sedentary behavior**							
Boddy et al. 2015, UK	ASD: N = 9, sample characteristics not reported separately for children with ASD	Parent reported diagnosis	All with ID	Actigraph, SED ≤100 CPM; ≥9h/day on weekday, ≥8h/day on weekend, ≥3 days	Total sedentary time	**ASD**: 428 (12)	3
MacDonald et al., 2011, USA	ASD: N = 72, range 9-18yrs, stratified by younger age group (9-11yrs) and older age group (12-19yrs), 76% boys	Study confirmed diagnosis (met Social Responsiveness Scale cutoff)	Younger age group: Mean IQ^c^ = 82.92, SD = 19.69; older age group: Mean IQ^c^ = 79.88, SD = 20.45	Actical, <100 CPM ≥10h/day	Total sedentary time	**ASD**: *Younger age group*: 667 (107); *Older age group*: 789 (114)	3
Tyler et al., 2014, USA	ASD: N = 17, ave age 12.6±2.3yrs, 53% boys; TD: N = 12, ave age 9.0±1.8yrs, 50% boys	Study confirmed diagnosis (met ADOS cutoff)	Verbal IQ mean = 60.1, SD = 25, range 16–110; Nonverbal IQ mean = 65.7, SD = 40, range 16–172	Actigraph, <150 CPM; Inclusion criteria not reported	Total sedentary time	**ASD**: 452 (101), **TD**: 369 (106) (p<0.001)	3
**Subjectively measured sedentary behavior**							
Chonchalya et al. 2011, Thailand	ASD: N = 65, ave age 2.6±0.7yrs, 78% boys; TD: N = 84, ave age 2.4±0.8yrs, 66% boys	Study confirmed diagnosis using DSM-IV-TR	Not reported^d^	Parent survey	Screen time (TV viewing)	**ASD**: 276 (115); **TD**: 124 (73) (p<0.0001)	3
Dreyer Gillette et al. 2015, USA	ASD: N = 900, range 10-17yrs, 84% boys; TD: N = 45000, range 10-17yrs, 50% boys	Parent reported diagnosis	Not reported	Parent survey	Screen time (TV/ video/ videogaming, computer/ cell phone/ electronics use)	% of sample engaging in specified hr/day screen time: *TV/ video/ videogaming*: **ASD**: 41% <1hr/d, 42% 1-4hr/d, 16% ≥4hr/d; **TD**: 41% <1hr/d, 45% 1-4hr/d, 15% ≥4hr/d; *Computer/ cell phone/ electronics use*: **ASD**: 20% never, 43% <1hr/d, 26% 1-4hr/d, 12% ≥4hr/d; **TD**: 9% never, 47% <1hr/d, 29% 1-4hr/d, 16% ≥4hr/d, (sig difference between % of ASD and TD children never engaging in computer/ cell phone/ electronics use only; p<0.01)	3
Engelhardt et al. 2013, USA	ASD: N = 49, ave age 11.8±2.7yrs, 100% boys; TD: N = 41, ave age 12.2±2.4yrs, 100% boys	Prior diagnosis. DSM-IV-TR Autistic Disorder (42%), Asperger’s Disorder (27%), PDD-NOS (31%)	N = 3 with IQ ≤70; remainder IQ >70	Parent survey	TV viewing, video-gaming	*TV viewing*: **ASD**: 138 (78), **TD**: 102 (54) (p = 0.03); *video-gaming*: **ASD**: 126 (78), **TD**: 72 (54); (p = 0.004)	3
Foran et al. 2012, USA	ASD: N = 174, ave age 10.3±0.4.3yrs, 86% boys	Parent reported diagnosis	Non-ID sample^e^	Parent survey	Screen time (video-gaming)	**ASD**: 86 (73)	3
Kuo et al. 2014, Canada	ASD: N = 91, ave age 14.8±1.9yrs, 81% boys	Parent reported diagnosis. Asperger’s syndrome (58%), Autistic Disorder (19%), PDD-NOS (18%)	7% with ID	Self-report survey	TV viewing, computer	*TV viewing*: **ASD**: 135 (116); *computer*: **ASD**: 295 (235)	3
Kuo et al. 2015, Canada	ASD: N = 29, ave age 15.1±2.3yrs, 90% boys; TD: N = 16, ave age 14.4±2.2yrs, 50% boys	Prior diagnosis. DSM-IV-TR	Not reported^f^	Parent report	TV viewing, video-gaming	*TV viewing*: **ASD**: 174 (162), **TD**: 228 (144), (not sig; p value not reported); *video-gaming*: **ASD**: 144 (150), **TD**: 90 (156), (not sig; p value not reported)	3
MacMullin et al. 2016, Canada	ASD: N = 139, ave age 11.7±3.6yrs, range 6-21yrs, 84% boys; TD: N = 172, ave age 12.3±4.0yrs, range 6-21yrs, 80% boys	Parent reported diagnosis. DSM-IV-TR. Autistic Disorder (52%), Asperger’s syndrome (63%), PDD-NOS, (11%)	Not reported	Parent survey	Device use (laptop, desktop, smart phone, iPad/iPod/ tablet)	*Boys*: **ASD**: 201 (62), **TD**: 187 (60); *Girls*: **ASD**: 191 (60), **TD**: 184 (47); (not sig; p value not reported)	3
Mazurek et al. 2013, USA	ASD: N = 202, ave age 12.1±2.8yrs, 84% boys; TD: N = 179, ave age 12.5±2.6yrs, 49% boys	Parent reported diagnosis. Autistic disorder (53.5), Asperger’s Disorder (27.2), PDD-NOS (17.3)	9.4% ≤70, 6.6% >70, 45% unknown	Parent survey	TV viewing, video-gaming, social media	*TV viewing (boys)*: **ASD**: 132 (90), **TD**: 114 (66); *TV viewing (girls)*: **ASD**: 144 (102), **TD**: 114 (60); *video-gaming (boys)*: **ASD**: 144 (102), **TD**: 96 (66); *video-gaming (girls)*: **ASD**: 108 (78), **TD**: 48 (54); *social media (boys)*: **ASD**: 12 (36), **TD**: 48 (66); *social media (girls)*: **ASD**: 18 (42), **TD**: 72 (72); (sig differences between ASD and TD children for video-gaming and social media only; p<0.001 for all)	3
Mazurek et al. 2013, USA	ASD: N = 56, ave age. 11.7±2.6yrs, 100% boys; TD: N = 41, ave age. 12.2±2.4yrs, 100% boys	Prior diagnosis. Autistic disorder (46%), Asperger’s Disorder (25%), PDD-NOS (29%)	N = 4 with IQ ≤70	Parent survey	Screen time (video-gaming)	**ASD**: 126 (78), **TD**: 72 (54) (p = 0.01)	3
Mazurek et al. 2013, USA	ASD: N = 169, ave age 12.1±2.8yrs, 100% boys	Parent reported diagnosis. Autistic Disorder (53%), Asperger’s Disorder (28%), PDD-NOS (19%)	9% with IQ ≤70	Parent survey	Screen time (video-gaming)	**ASD**: 144 (102)	3
Must et al. 2014, USA	ASD: N = 53, ave age 6.6±2.1yrs, 83% boys; TD: N = 58, ave age 6.7±2.4yrs, 78% boys	Study confirmed diagnosis (met ADI-R cutoff)	Mean IQ^g^ = 85.8, SD = 22.1	Parent survey	Screen time, total sedentary time	*Screen time (weekday)*: **ASD**: 150 (13), **TD**: 96 (13); *screen time (weekend day)*: **ASD**: 234 (19), **TD**: 186 (19); *total sedentary time (weekday)*: **ASD**: 312 (20), **TD**: 252 (19); *total sedentary time (weekend day)*: **ASD**: 438 (26), **TD**: 414 (25); (sig differences between ASD and TD children for all except weekend day sedentary time; p<0.05)	3
Must et al. 2015, USA	ASD: N = 53, ave age 6.6±2.1yrs, 83% boys; TN: N = 58, ave age 6.7±2.4yrs, 78% boys	Study confirmed diagnosis (met ADI-R cutoff)	Mean IQ = 85.8, SD = 22.1^g^	Parent survey	Not reported	Not reported	3
Orsmond et al. 2011, USA	ASD: N = 103, ave age 17.8±2.2yrs (range 12-21yrs), 76% boys	Prior diagnosis (met ADI-R met)	56% with ID	Parent survey	TV viewing; computer	*TV viewing*: **ASD**: 136 (131); computer: **ASD**: 99 (98)	3
Shane et al. 2008, USA	ASD: N = 89, ave age—all<18yrs, 86% boys	Not reported	Parents were not asked to report	Parent survey	Screen time (TV, video, DVD, computer, games + educational)	**ASD**: % of children engaging in specified hr/day on *weekdays*: *TV*: 52% 0–1 hr/day, 27% 1–3 hr/day, 23% 3+ hr/day; *video/DVD*: 36% 0–1 hr/day, 39%, 1–3 hr/day, 24% 3+ hr/day; *computer games*: 64% 0–1 hr/day, 31% 1–3 hr/day, 3% 3+ hr/day; *educational software*: 76% 0–1 hr/day, 21% 1–3 hr/day, 2% 3+ hr/day; % of children engaging in specified hr/day on *weekend days*: *TV*: 62% 0–1 hr/day, 23% 1–3 hr/day, 16% 3+ hr/day; *video/DVD*: 54% 0–1 hr/day, 36%, 1–3 hr/day, 9% 3+ hr/day; *computer games*: 72% 0–1 hr/day, 24% 1–3 hr/day, 3% 3+ hr/day; *educational software*: 81% 0–1 hr/day, 17% 1–3 hr/day, 1% 3+ hr/day	3
Soden et al. 2012, USA	ASD: N = 26, ave age 13.3±2.8yrs, 81% boys	Prior diagnosis (DSM-IV-TR) N = 9 Autistic disorder, N = 6 Asperger’s disorder, N = 11 PDD-NOS	Not reported	Parent survey	Screen time	**ASD**: 251 (234)	3

^a^ All studies were cross-sectional, except Kuo et al., 2015[56] and Orsmond et al. 2011[32];

^b^ 1 = strong quality/low risk of bias, 2 = moderate quality/risk of bias, 3 = weak quality/high risk of bias;

^c^ Measured using 2 subtests from the WASI;

^d^ Delay between language development level and chronological age reported to be -1.22 years;

^e^ This subgroup was reported not to have comorbid ID;

^f^ 35% reported to be in a special education class;

^g^ Measured using DAS General Conceptual Ability Score

Abbreviations: ADI-R—revised Autism Diagnostic Interview; ADOS—Autism Diagnostic Observation Schedule; ave age—average age; ASD—children with Autism Spectrum disorder; DSM-IV-TR—Diagnostic and Statistical Manual of Mental Disorders; ID—intellectual disability; IQ—intelligence quotient; min—minutes; PDD-NOS—pervasive developmental disorder not otherwise specified; SD—standard deviation; sig—significant; TD—typically developing children; TV—television; yrs—years

Six of the 18 SB studies had study-confirmed ASD diagnosis [37, 38, 44, 46, 52, 53], five used prior reports of diagnosis [32, 47, 51, 54, 56], and seven used parent reports [39, 43, 48–50, 55, 57]. Six studies used DSM-IV criteria [46, 47, 53–56]. One study included participants only with ID [43], one included participants only without ID [48], and six included participants with and without ID [32, 47, 49–51, 57]. Four studies reported the mean IQ of participants, ranging from 60.1 to 85.8 [37, 38, 44, 52], and five studies did not report ID or IQ [39, 46, 54–56].

All 18 studies reporting SB outcomes were classified as having high risk of bias. All studies reporting SB outcomes used an appropriate type of analysis for the study design (S5 Table).

### Sedentary behavior—Prevalence

Twelve studies reported SB as screen time which incorporated activities such as TV watching, video gaming, computer and social media use [32, 46–52, 54–57]. Two studies reported the use of smart phone technology [39, 55]; one reported on digital tablet use [55]. However, those studies only reported time using those devices as part of a variable which included time using other devices (e.g. laptop) also. Four studies reported total sedentary time (i.e. measured objectively) [37, 38, 43]. Two studies reported sedentary time as a percentage of the whole day, thus these data could not be compared with other studies [39, 53]. The average number of minutes/day of total screen time ranged from 86 to 430 (average 231 min/day) and the average number of minutes/day of total sedentary time ranged from 312 to 789 (average 514 min/day).

Six studies [39, 46, 49, 51, 55, 56] reported the prevalence of SB for both ASD and TD samples; only two studies reported significant differences in SB between ASD and TD children. Chonchaiya et al. [46] found that children with ASD spent significantly more time in screen time (4.6 vs. 2.6 hours/day) and fewer complied with screen time recommendations of less than two hours per day (6% vs 44%) compared to their TD counterparts. In contrast, Dreyer Gillette et al. [39] found that a significantly higher percent of children with ASD (19.8%) never used a computer/cell phone/hand held video games/other electronic devices compared with their TD counterparts (8.7%). However, that study found no difference in children’s use of TV/video/video games, nor in the number of hours children used computer/cell phone/hand held video games/other electronic devices.

### Sedentary behavior—Correlates

Eleven studies reported potential correlates of SB (Table 5), with 23 different correlates examined: 15 were child variables; eight were familial variables; no physical environment variables were reported. Only two variables were reported in sufficient studies to determine an overall association. Age and sex showed an inconsistent association with SB across studies.

**Table 5:** Correlates of sedentary behavior among children diagnosed with Autism Spectrum Disorder.

Correlate	Associated with SB (reference)	Association (+/-)	Not associated with SB (reference)	Summary coding for row (n/N for row, %)	Summary code for association (-/+)
**Child Variables**					
Age	Foran [48], MacDonald [37], Mazurek [50]	+	Engelhardt [47], Kuo [57], Orsmond [32]	3/6 (50)	??
Sex	Foran [48] (boys only), Kuo [57] (boys only, video-gaming only)	+	MacDonald [37], Orsmond [32], MacMullin [55]	2/5(40)	??
Race			Mazurek [51]	0/1 (0)	0
BMI z-score	Must [52] (weekend SB and weekend screen time)	+	Must [52] (weekday SB and weekday screen time)	1/2(50)	?
Average sleep			Engelhardt [47]	0/1 (0)	0
Physical activity	Must [52] (weekday SB and weekday screen time)	-	Must [52] (weekend SB and weekend screen time)	1/2(50)	?
ASD symptom severity	Engelhardt [47]	+	Kuo [57], MacDonald [37]	1/2(50)	?
Comorbidities	Orsmond [32]	-	Kuo [57], Mazurek [50]	1/3(33)	0
IQ			MacDonald [37], Mazurek [51]	0/2 (0)	0
Addiction (video use)	Mazurek [50]	+			
Inattention			Mazurek [50]	0/1 (0)	0
Hyperactivity			Mazurek [50]	0/1 (0)	0
Verbal communication impairment	Orsmond [32] (TV and computer use)	-		1/1 (100)	-
Maladaptive behaviors			Orsmond [32]	0/1 (0)	0
Oppositional defiant behvior			Mazurek [50]	0/1 (0)	0
**Familial variables**					
Parental education			Kuo [57]	0/1 (0)	0
Maternal education			Orsmond [32]	0/1 (0)	0
Parental employment			Kuo [57]	0/1 (0)	0
Family income	Kuo [57] (video-gaming only)		Mazurek [51]	1/2 (50)	?
Parent marital status	Orsmond [32] (married, computer use)	+	Mazurek [51]	1/2 (50)	?
Family size/number of siblings	Orsmond [32] (TV)	-	Engelhardt [47]	1/2 (50)	?
Perceived parental barriers	Mus t[44]	+		1/1 (100)	+
Parental mediation of screen time	Kuo [56] (social mediation and active mediation)	+	Kuo [56] (restrictive mediation for TV), Kuo [56] (restrictive mediation, social mediation, active mediation—video gaming)	1/3 (33)	0

## Discussion

Prevalence rates and correlates of PA and SB among children diagnosed with ASD, and how these potentially differ from TD children, largely remains unknown. In this review, estimates of time in MVPA and screen time per day, and compliance with MVPA and SB recommendations, varied for children with ASD and typically developing children between studies. Despite the substantial variations in estimates of time spent in PA and screen time for ASD and TD children, children with ASD seem to spend more time in screen time (and in turn total SB) and less time in PA than TD children (26–430 mins/day in screen time for children with ASD, compared with 72–318 mins/day in screen time for typically developing children [54, 57, 59, 64]). It is well documented that estimates of behaviors such as PA and SB, and subsequently estimates of compliance with recommendations, vary, based on method of data collection (e.g. survey, accelerometry), data management methods [58–60] and other factors such as season [61]. Nonetheless, PA appears low and SB high among children with ASD (when mean minutes per day is used to compare children) and targeted interventions may be warranted once potential correlates are identified.

Of the 47 potential correlates examined in the studies in this review (24 for PA and 23 for SB), most (53%) were classified as child variables. Only two child variables, age and sex, were reported sufficient times across studies to allow overall assumptions to be made. Consistent with the literature among TD children, this review found negative associations between PA and age or school level/grade [62, 63]. That is, as children’s age/school level/grade increases, their level of PA decreases. This suggests that PA interventions for children with ASD should be implemented as early as possible to maximize PA while children are young and curb potential age-associated decreases. In contrast to current literature for TD children, this review found an overall inconsistent association of sex with PA among children with ASD. Among typically developing populations, boys are consistently more active than girls [26, 64–66]. The null association reported may have been influenced by the demographics of the participants in the included papers. The two studies which reported that boys were more active than girls had participants, on average, younger than 10 years old. However, for the other two studies where no association between sex and physical activity was identified, the average age of the participants was several years older. As children’s age increases, it is feasible that the influence of sex is less defined among children with ASD. Nonetheless, the small number of studies reporting sex as a potential correlate is likely to limit the opportunity to identify any individual variable as a correlate and further research is needed to more comprehensively understand whether or not boys with ASD are more active than girls with ASD.

In terms of SB, in TD children mixed associations have been reported for age and sex, some reporting strong, consistent associations [67] and others inconsistent associations [24, 64, 68, 69]. In the current review, inconsistent associations of age and sex with SB were reported [70–73]. The relatively small number of studies included in this review may have contributed to the inconsistent associations (six studies reported associations between SB and age and five studies between SB and sex). Furthermore, the small sample sizes for most of the studies included in this review may mean studies are not powered to detect associations. Some studies included only a narrow age range for their samples (for instance, 2.6 years ±0.7 years [46]) and other characteristics may similarly be relatively homogenous given the small samples. Therefore, it is possible that sample characteristics may have insufficient diversity for associations to be identified.

A number of child-related ‘ASD specific’ potential correlates such as ASD symptom severity [37, 45, 47, 57], maladaptive behaviors [32], and psychotropic medication (taking or not taking) [30] were investigated; none were investigated in four or more studies for either PA or SB. Although these variables were not strongly associated, they still may be important correlates to consider in the design of future interventions. There is a growing awareness that ASD is a heterogeneous disorder, with many complexities and comorbidities [74] which give rise to large variations in health and well-being: it is often the type and nature of associated comorbidities and health-related factors which may influence children’s outcomes, possibly including reduced opportunity for PA [75]. It is therefore essential to report such comorbidities in studies to determine their relative contribution to health behaviors as well as the contribution PA and SB may have in supporting positive health outcomes. Given the prevalence of ASD comorbidities and other ‘ASD specific’ potential correlates, it may be that specialized facilitators are the most appropriate personnel to implement interventions. Such personnel would likely have a greater understanding of the complexities of ASD and how different comorbidities may affect PA and SB levels.

The correlates of PA and SB for TD children have been thoroughly evaluated and reported, for instance: [24, 25, 64, 67, 76–80]. While some reviews include a broad range of correlates, such as individual, social and physical environment correlates [25, 26, 64, 67, 81], others have focused specifically on one domain or construct (e.g. physical environment, neighborhood safety [82, 83]). Reviews in TD children also typically focus on a narrower age range than this review does (e.g., preschoolers [24, 80], adolescents [78, 84]), and correlates vary across reviews. As in this review, reviews of the correlates of TD children’s PA and SB typically identify many variables which have been investigated in only one or two studies. It is therefore difficult to provide a clear and synthesized overview of correlates in TD children against which to compare the correlates in children with ASD. However, across reviews in TD children, boys appear to be more active than girls [24, 64], younger children are more active than older children [62, 63], children with active parents are more active [24, 64, 85], and children who spend more time outdoors are more active [24, 64]. Parental rules [85], family TV viewing [67] and age [73] may be associated with SB in TD children.

Given the dearth of PA and SB interventions for children with ASD [18], interventions specific for children with ASD may be needed. The content and behavior change strategies to be included in such interventions require further research. Studies investigating prevalence of SB in children with ASD, which include the full range of electronic devices (e.g. computer, digital tablets) are required. In this review no studies of the time children with ASD use digital tablets met the inclusion criteria. With increasing use of these devices in children with ASD for entertainment and education [86–88], and strong acceptance by parents and educators [89], studies investigating this behavior are warranted.

### Limitations of existing data and subsequent recommendations

This is the first review to summarize prevalence of PA and SB among children with ASD and to report on PA and SB correlates. Results and recommendations should be considered in light of the following limitations:

1. Sample sizes: Studies in this review generally included small samples. Mean sample sizes for PA and SB studies were 41 and 82, respectively, after the one very large study was removed as an outlier. SB studies, on average, included twice as many participants with ASD as PA studies and all studies involved more boys than girls (consistent with more boys (4.3:1) being diagnosed with ASD than girls [90, 91]). Ensuring samples are sufficient to provide meaningful results is essential. It is therefore recommended that future studies include a power calculation to ensure they recruit a sample of sufficient size to be able to identify associations should they exist and to provide meaningful results. Furthermore, investigating prevalence rates and correlates for different age groups and both sexes would be helpful to inform future interventions.
2. Age of participants: many of the studies included in this review utilized samples with large ranges in participant ages and reported aggregated data across all children. This may be necessary with small sample sizes. Nonetheless, it is recommended that future studies focus on a narrower age range where children are typically at the same or similar developmental stage (e.g. 3–5 years, 5–11 years, 12–18 years), and exposed to similar environments (e.g. preschool, primary school, secondary school) to allow more meaningful and potentially useful results to be reported.
3. Number of correlates: In this review, few correlates have been investigated in sufficient studies to draw consistent conclusions. It is recommended that future studies endeavor to measure as broad a range of potential correlates as possible, as studies in TD children show that correlates exist across all domains of the ecological model [24, 25, 64, 92–95]. This may also be the case among children with ASD. Including a broad range of correlates allows for the relative strength of associations to be examined when other influences are controlled for and aids future intervention development to ensure efficacious outcomes. Although variables included will be specific to each study to meet the intended aims, suggestions include: age, sex, ASD symptom severity, perceived parental barriers, parental self-efficacy, and characteristics of the neighborhood environment which may be supportive of physical activity. Further, a number of potentially important correlates remain to be examined: no studies examined motor competence [96], preference for sedentary/active opportunities [97], access to facilities or neighborhood environment characteristics [98, 99], which may support or constrain behaviors.
4. Consistent reporting of outcomes: In this review, reporting of data within studies was inconsistent. For example, different types of SB were reported in each study: some reported time watching television [46] or video-gaming [49, 50]; others reported television viewing and video-gaming or computer use [32, 47]. No studies were identified which included specific SB other than screen behaviors, such as reading or doing craft. Reporting varied between studies, making comparisons difficult: some reported prevalence rates over seven days [33, 34, 38, 43]; others only reported weekday and/or weekend prevalence [40, 42]. Most studies reporting SB outcomes used proxy-report questionnaires: only five reported validity and reliability data for those questionnaires, raising concerns regarding instrument accuracy [32, 38, 43, 44, 52]. It is recommended that future studies, where feasible, assess physical activity and sedentary behavior/screen time using reliable and validated instruments, ideally using objective measures and reporting over a seven-day period.
5. ASD diagnosis: The majority of studies did not confirm ASD diagnosis of the children and relied on prior or parent-report. DSM criteria were seldom reported as confirmation of clinical diagnosis. Many studies included children with comorbid ID. It is unclear whether PA and SB are impacted by ASD symptom severity, having comorbid ID or compounded by the presence of both. Thus, it is recommended that future studies consistently report children’s diagnosis of ASD, in line with DSM diagnostic criteria and take into consideration common co-morbidities for this clinical populations.
6. Risk of bias: The risk of bias of the studies in this review was overwhelmingly high; this is important as it aids in comparison [100] and should be considered in future research in this population. It is possible that studies included in this review were methodologically robust but were rated as having high risk of bias due to inadequate reporting. It is recommended that reporting details of studies in line with existing statements (e.g. STROBE [101], CONSORT [102]) should be adhered to so study risk of bias, rather than poor reporting, can be accurately assessed.

## Conclusions

This review highlights generally lower levels of PA and higher levels of SB among children with ASD compared to TD children [103]. Furthermore, this review highlights that age is consistently inversely associated, and sex inconsistently associated with physical activity. Age and sex are inconsistently associated with sedentary behavior. For optimal physical activity and sedentary behavior levels, this review supports the notion that children with ASD could potentially benefit from lifestyle modifications that promote increased physical activity and sedentary behavior. Additionally, given the complexity of ASD, studies investigating additional correlates of ASD would be beneficial. Cohort studies would be beneficial in providing a stronger level of evidence and showing temporal associations.

## Supporting information

S1 Table Completed PRISMA checklist. (DOCX)

S2 Table Data extraction table for the physical activity papers. (XLSX)

S3 Table Data extraction table for the sedentary behavior papers. (XLSX)

S4 Table Risk of bias ^a^ for papers reporting physical activity outcomes. ^a^ 1 = strong quality/low risk of bias, 2 = moderate quality/risk of bias, 3 = weak quality/high risk of bias.
(DOCX)

S5 Table Risk of bias ^a^ for papers reporting sedentary behavior outcomes. ^a^ 1 = strong quality/low risk of bias, 2 = moderate quality/risk of bias, 3 = weak quality/high risk of bias.
(DOCX)

## References

[1] Hinkley T, Teychenne M, Downing KL, Ball K, Salmon J, Hesketh KD. Early childhood physical activity, sedentary behaviors and psychosocial well-being: a systematic review. Prev Med. 2014;62: 182–92. 10.1016/j.ypmed.2014.02.007 24534461

[2] Carson V, Hunter S, Kuzik N, Wiebe SA, Spence JC, Friedman A, et al Systematic review of physical activity and cognitive development in early childhood. J Sci Med Sport. 2015;19: 573–8. 10.1016/j.jsams.2015.07.011 26197943

[3] Carson V, Kuzik N, Hunter S, Wiebe SA, Spence JC, Friedman A, et al Systematic review of sedentary behavior and cognitive development in early childhood. Prev Med. 2015;78: 115–22. 10.1016/j.ypmed.2015.07.016 26212631

[4] Timmons BW, Leblanc AG, Carson V, Connor Gorber S, Dillman C, Janssen I, et al Systematic review of physical activity and health in the early years (aged 0–4 years). Appl Physiol Nutr Metab. 2012;37: 773–92. 10.1139/h2012-070 22765840

[5] Leblanc AG, Spence JC, Carson V, Connor Gorber S, Dillman C, Janssen I, et al Systematic review of sedentary behaviour and health indicators in the early years (aged 0–4 years). Appl Physiol Nutr Metab. 2012;37: 753–72. 10.1139/h2012-063 22765839

[6] Janssen I, Leblanc AG. Systematic review of the health benefits of physical activity and fitness in school-aged children and youth. Int J Behav Nutr Phys Act. 2010;7: 40 10.1186/1479-5868-7-40 20459784 PMC2885312

[7] Tremblay MS, LeBlanc AG, Kho ME, Saunders TJ, Larouche R, Colley RC, et al Systematic review of sedentary behaviour and health indicators in school-aged children and youth. Int J Behav Nutr Phys Act. 2011;8: 98 10.1186/1479-5868-8-98 21936895 PMC3186735

[8] Active Healthy Kids Australia. Is Sport Enough? The 2014 Active Healthy Kids Australia Report Card on Physical Activity for Children and Young People. Adelaide, South Australia: Active Healthy Kids Australia, 2014.

[9] Active Healthy Kids Scotland. The 2013 Active Healthy Kids Scotland Report Card. Glasgow: Active Healthy Kids Scotland, 2013.

[10] Healthy Active Kids New Zealand. The New Zealand Physical Activity Report Card for Children and Youth. Auckland, New Zealand: National Institute for Health Innovation, University of Auckland, 2014.

[11] National Physical Activity Plan Alliance. United States Report Card on Physical Activity for Children and Youth. Columbia SC: National Physical Activity Plan Alliance, 2014.

[12] ParticipACTION. The Biggest Risk is Keeping Kids Indoors. The 2015 ParticipACTION Report Card on Physical Activity for Children and Youth. Toronto: ParticipACTION, 2015.

[13] Stratton G, Williams C, Taylor S, Jones A, MacKintosh K, Frost M, et al. Active Healthy Kids Report Card—Wales. Swansea: Swansea University, 2014.

[14] American Psychiatric Association. Diagnostic & Statistical Manual of Mental Disorders. 5 ed Washington DC: American Psychiatric Publishing; 2013.

[15] Elsabbagh M, Divan G, Koh YJ, Kim YS, Kauchali S, Marcin C, et al Global prevalence of autism and other pervasive developmental disorders. Autism Res. 2012;5: 160–79. 10.1002/aur.239 22495912 PMC3763210

[16] Randall M, Sciberras E, Brignell A, Ihsen E, Efron D, Dissanayake C, et al Autism spectrum disorder: Presentation and prevalence in a nationally representative Australian sample. Aust N Z J Psychiatry. 2016;50: 243–53. 10.1177/0004867415595287 26282446

[17] Zablotsky B, Black LI, Maenner MJ, Schieve LA, Blumberg SJ. Estimated Prevalence of Autism and Other Developmental Disabilities Following Questionnaire Changes in the 2014 National Health Interview Survey. Natl Health Stat Report. 2015: 1–20. 26632847

[18] Fournier KA, Hass CJ, Naik SK, Lodha N, Cauraugh JH. Motor coordination in autism spectrum disorders: a synthesis and meta-analysis. J Autism Dev Disord. 2010;40: 1227–40 14 p. 10.1007/s10803-010-0981-3 20195737

[19] Hill AP, Zuckerman KE, Fombonne E. Obesity and Autism. Pediatrics. 2015;136: 1051–61. 10.1542/peds.2015-1437 26527551 PMC4657601

[20] Bhat AN, Landa RJ, Galloway JC. Current perspectives on motor functioning in infants, children, and adults with autism spectrum disorders. Phys Ther. 2011;91: 1116–29. 10.2522/ptj.20100294 21546566

[21] Pan CY, Tsai CL, Hsieh KW. Physical Activity Correlates for Children With Autism Spectrum Disorders in Middle School Physical Education. Res Q Exercise Sport. 2011;82: 491–8. 10.1080/02701367.2011.10599782 21957708

[22] Moher D, Liberati A, Tetzlaff J, Altman DG, Group P. Preferred reporting items for systematic reviews and meta-analyses: the PRISMA statement. J Clin Epidemiol. 2009;62: 1006–12. 10.1016/j.jclinepi.2009.06.005 19631508

[23] World Health Organization. Global Strategy on Diet, Physical Activity and Health: Recommended levels of physical activity for children aged 5–17 years 2016 [updated 16 February 2016. http://www.who.int/dietphysicalactivity/factsheet_young_people/en/.

[24] Hinkley T, Crawford D, Salmon J, Okely AD, Hesketh K. Preschool children and physical activity: a review of correlates. Am J Prev Med. 2008;34: 435–41. 10.1016/j.amepre.2008.02.001 18407012

[25] Hinkley T, Salmon J, Okely AD, Trost SG. Correlates of sedentary behaviours in preschool children: a review. Int J Behav Nutr Phys Act. 2010;7: 66 10.1186/1479-5868-7-66 20825682 PMC2945987

[26] Ridgers ND, Salmon J, Parrish AM, Stanley RM, Okely AD. Physical activity during school recess: a systematic review. Am J Prev Med. 2012;43: 320–8. 10.1016/j.amepre.2012.05.019 22898126

[27] Bronfenbrenner U. The Ecology of Human Development: Experiments by nature and design. Cambridge: Harvard University Press; 1979.

[28] National Collaborating Centre for Methods and Tools. Quality Assessment Tool for Quantitative Studies2008 [Updated 13 April, 2010]. http://www.nccmt.ca/registry/view/eng/14.html.

[29] Bandini LG, Gleason J, Curtin C, Lividini K, Anderson SE, Cermak SA, et al Comparison of physical activity between children with autism spectrum disorders and typically developing children. Autism. 2013;17: 44–54. 10.1177/1362361312437416 22807562 PMC3690470

[30] Memari AH, Ghaheri B, Ziaee V, Kordi R, Hafizi S, Moshayedi P. Physical activity in children and adolescents with autism assessed by triaxial accelerometry. Pediatr Obes. 2012;8: 150–8. 10.1111/j.2047-6310.2012.00101.x 23042790

[31] Obrusnikova I, Cavalier AR. Perceived barriers and facilitators of participation in after-school physical activity by children with autism spectrum disorders. J Dev Phys Disabil. 2011;23: 195–211.

[32] Orsmond GI, Kuo H-Y. The daily lives of adolescents with an autism spectrum disorder: Discretionary time use and activity partners. Autism. 2011;15: 579–99. 10.1177/1362361310386503 21697194 PMC3572828

[33] Pan CY, Frey GC. Identifying physical activity determinants in youth with autism spectrum disorders. Res Q Exercise Sport. 2005;76: A116–A7.

[34] Pan C-Y, Frey GC. Physical Activity Patterns in Youth with Autism Spectrum Disorders. J Autism Dev Disord. 2006;36: 597–606. 10.1007/s10803-006-0101-6 16652237

[35] Pan C-Y, Tsai C-L, Hsieh K-W, Chu C-H, Li Y-L, Huang S-T. Accelerometer-determined physical activity among elementary school-aged children with autism spectrum disorders in Taiwan. Res Autism Spect Disord. 2011;5: 1042–52.

[36] Sandt DDR, Frey GC. Comparison of Physical Activity Levels Between Children With and Without Autistic Spectrum Disorders. Adapt Phys Act Q. 2005;22: 146.

[37] Macdonald M, Esposito P, Ulrich D. The physical activity patterns of children with autism. BMC Res Notes. 2011;4: 422-. 10.1186/1756-0500-4-422 22008607 PMC3213672

[38] Tyler K, MacDonald M, Menear K. Physical activity and physical fitness of school-aged children and youth with autism spectrum disorders. Autism Res Treat. 2014;2014: 312163-. 10.1155/2014/312163 25309753 PMC4182001

[39] Dreyer Gillette ML, Borner KB, Nadler CB, Poppert KM, Odar Stough C, Swinburne Romine R, et al Prevalence and Health Correlates of Overweight and Obesity in Children with Autism Spectrum Disorder. J Dev Behav Pediatr. 2015;36: 489–96. 26166285 10.1097/DBP.0000000000000198

[40] Tatsumi Y, Mohri I, Shimizu S, Tachibana M, Ohno Y, Taniike M. Daytime physical activity and sleep in pre-schoolers with developmental disorders. J Paediatr Child Health. 2014. 10.1111/jpc.12725 25187236

[41] Ayvazoglu NR, Kozub FM, Butera G, Murray MJ. Determinants and challenges in physical activity participation in families with children with high functioning autism spectrum disorders from a family systems perspective. Res Dev Disabil. 2015;47: 93–105. 10.1016/j.ridd.2015.08.015 26368652

[42] Wachob D, Lorenzi DG. Brief Report: Influence of Physical Activity on Sleep Quality in Children with Autism. J Autism Dev Disord. 2015;45: 2641–6. 10.1007/s10803-015-2424-7 25791123

[43] Boddy LM, Downs SJ, Knowles ZR, Fairclough SJ. Physical activity and play behaviours in children and young people with intellectual disabilities: A cross-sectional observational study. Sch Psychol Int. 2015;36: 154–71.

[44] Must A, Phillips S, Curtin C, Bandini LG. Barriers to Physical Activity in Children With Autism Spectrum Disorders: Relationship to Physical Activity and Screen Time. J Phys Act Health. 2015;12: 529–34. 10.1123/jpah.2013-0271 25920014 PMC4490003

[45] Memari AH, Panahi N, Ranjbar E, Moshayedi P, Shafiei M, Kordi R, et al Children with Autism Spectrum Disorder and Patterns of Participation in Daily Physical and Play Activities. Neurol Res Int. 2015;2015. 10.1155/2015/531906 PMC4485548 26171247

[46] Chonchaiya W, Nuntnarumit P, Pruksananonda C. Comparison of television viewing between children with autism spectrum disorder and controls. Acta Paediatr. 2011;100: 1033–7. 10.1111/j.1651-2227.2011.02166.x 21244489

[47] Engelhardt CR, Mazurek MO, Sohl K. Media use and sleep among boys with autism spectrum disorder, adhd, or typical development. Pediatrics. 2013;132: 1081–9. 10.1542/peds.2013-2066 24249825

[48] Foran AC, Cermak SA. Active and Traditional Videogame Ownership and Play Patterns Among Youths With Autism Spectrum Disorders. Palaestra. 2013;27: 42–8.

[49] Mazurek MO, Engelhardt CR. Video game use in boys with autism spectrum disorder, ADHD, or typical development. Pediatrics. 2013;132: 260–6. 10.1542/peds.2012-3956 23897915

[50] Mazurek MO, Engelhardt CR. Video game use and problem behaviors in boys with autism spectrum disorders. Res Autism Spect Disord. 2013;7: 316–24.

[51] Mazurek MO, Wenstrup C. Television, video game and social media use among children with ASD and typically developing siblings. J Autism Dev Disord. 2013;43: 1258–71. 10.1007/s10803-012-1659-9 23001767

[52] Must A, Phillips SM, Curtin C, Anderson SE, Maslin M, Lividini K, et al Comparison of sedentary behaviors between children with autism spectrum disorders and typically developing children. Autism. 2014;18: 376–84. 10.1177/1362361313479039 24113339 PMC4152822

[53] Shane HC, Albert PD. Electronic screen media for persons with autism spectrum disorders: Results of a survey. J Autism Dev Disord. 2008;38: 1499–508. 10.1007/s10803-007-0527-5 18293074

[54] Soden SE, Garrison CB, Egan AM, Beckwith AM. Nutrition, physical activity, and bone mineral density in youth with autistic spectrum disorders. J Dev Behav Pediatr. 2012;33: 618–24. 10.1097/DBP.0b013e318260943c 23027134

[55] MacMullin JA, Lunsky Y, Weiss JA. Plugged in: Electronics use in youth and young adults with autism spectrum disorder. Autism. 2016;20: 45–54. 10.1177/1362361314566047 25694586

[56] Kuo MH, Magill-Evans J, Zwaigenbaum L. Parental mediation of television viewing and videogaming of adolescents with autism spectrum disorder and their siblings. Autism. 2015;19: 724–35. 10.1177/1362361314552199 25336095

[57] Kuo MH, Orsmond GI, Coster WJ, Cohn ES. Media use among adolescents with autism spectrum disorder. Autism. 2014;18: 914–23. 10.1177/1362361313497832 24142797

[58] Cliff DP, Reilly JJ, Okely AD. Methodological considerations in using accelerometers to assess habitual physical activity in children aged 0–5 years. J Sci Med Sport. 2009;12: 557–67. 10.1016/j.jsams.2008.10.008 19147404

[59] Beets MW, Bornstein D, Dowda M, Pate RR. Compliance with national guidelines for physical activity in U.S. preschoolers: measurement and interpretation. Pediatrics. 2011;127: 658–64. 10.1542/peds.2010-2021 21422082 PMC3387888

[60] Mâsse LC, Fuemmeler BF, Anderson CB, Matthews CE, Trost SG, Catellter DJ, et al Accelerometer Data Reduction: A Comparison of Four Reduction Algorithms on Select Outcome Variables. Med Sci Sport Exer. 2005;37: S544–S54. 10.1249/01.mss.0000185674.09066.8a 16294117

[61] Kolle E, Steene-Johannessen J, Andersen LB, Anderssen SA. Seasonal variation in objectively assessed physical activity among children and adolescents in Norway: A cross-sectional study. Int J Behav Nutr Phys Act. 2009;6. 10.1186/1479-5868-6-36 PMC2711042 19563650

[62] Czerwinski F, Finne E, Kolip P, Bucksch J. Individual and school level correlates of moderate to vigorous physical activity among school-children in Germany—a multi-level analysis. BMC public health. 2015;15: 393–1 p. 10.1186/s12889-015-1715-4 25928443 PMC4423129

[63] Dumith SC, Gigante DP, Domingues MR, Kohl HW 3rd. Physical activity change during adolescence: a systematic review and a pooled analysis. Int J Epidemiol. 2011;40: 685–98. 10.1093/ije/dyq272 21245072

[64] Sallis JF, Prochaska JJ, Taylor W. A review of correlates of physical activity of children and adolescents. Med Sci Sport Exer. 2000;32: 963–75. 10.1097/00005768-200005000-00014 10795788

[65] Hinkley T, Crawford D, Salmon J, Okely AD, Hesketh K. Preschool children and physical activity: a review of correlates. Am J Prev Med. 2008;34: 435–41. 10.1016/j.amepre.2008.02.001 18407012

[66] Bingham DD, Costa S, Hinkley T, Shire KA, Clemes SA, Barber SE. Physical Activity During the Early Years: A Systematic Review of Correlates and Determinants. Am J Prev Med. 2016;51: 384–402. 10.1016/j.amepre.2016.04.022 27378255

[67] Hoyos Cillero I, Jago R. Systematic review of correlates of screen-viewing among young children. Prev Med. 2010;51: 3–10. 10.1016/j.ypmed.2010.04.012 20417227

[68] Hinkley T, Salmon J, Okely AD, Crawford D, Hesketh K. Preschoolers' physical activity, screen time, and compliance with recommendations. Med Sci Sport Exer. 2012;44: 458–65. 10.1249/MSS.0b013e318233763b 21900847

[69] Gorely T, Marshall SJ, Biddle SJH. Couch kids: correlates of television viewing among youth. Int J Behav Med. 2004;11: 152–63 12 p. 10.1207/s15327558ijbm1103_4 15496343

[70] Anderson SE, Economos CD, Must A. Active play and screen time in US children aged 4 to 11 years in relation to sociodemographic and weight status characteristics: a nationally representative cross-sectional analysis. BMC public health. 2008;8: (22 October 2008)-(22 October). 10.1186/1471-2458-8-366 PMC2605460 18945351

[71] Herman KM, Sabiston CM, Mathieu M-E, Tremblay A, Paradis G. Correlates of sedentary behaviour in 8- to 10-year-old children at elevated risk for obesity. Appl Phys Nutr Metab. 2015;40: 10–9 p. 10.1139/apnm-2014-0039 25415850

[72] LeBlanc AG, Katzmarzyk PT, Barreira TV, Broyles ST, Chaput J-P, Church TS, et al Correlates of Total Sedentary Time and Screen Time in 9–11 Year-Old Children around the World: The International Study of Childhood Obesity, Lifestyle and the Environment. Plos One. 2015;10: e0129622–e. 10.1371/journal.pone.0129622 26068231 PMC4465981

[73] Temmel CSD, Rhodes R. Correlates of Sedentary Behaviour in Children and Adolescents Aged 7–18: A Systematic Review. Health Fit J Canada. 2013;6: 81.

[74] Rinehart N, McGillivray J, Kirkovski M, Williams K. Autism Spectrum Disorders In: Rinehart N, Bradshaw J, Enticott P, editors. Developmental Disorders of the Brain: Taylor and Francis, UK; under review.

[75] Gillberg C. The ESSENCE in child psychiatry: Early Symptomatic Syndromes Eliciting Neurodevelopmental Clinical Examinations. Res Dev Disabil. 2010;31: 1543–51. 10.1016/j.ridd.2010.06.002 20634041

[76] Tremblay L, Boudreau-Larivière C, Cimon-Lambert K. Promoting Physical Activity in Preschoolers: A Review of the Guidelines, Barriers, and Facilitators for Implementation of Policies and Practices. Can Psychol. 2012;53: 280–90.

[77] Mitchell J, Skouteris H, McCabe M, Ricciardelli LA, Milgrom J, Baur LA, et al Physical activity in young children: A systematic review of parental influences. Early Child Dev Care. 2012;182: 1411–37.

[78] Ferreira I, van der Horst K, Wendel-Vos W, Kremers S, van Lenthe FJ, Brug J. Environmental correlates of physical activity in youth—a review and update. Obes Rev. 2007;8: 129–54. 10.1111/j.1467-789X.2006.00264.x 17300279

[79] Xu H, Wen LM, Rissel C. Associations of parental influences with physical activity and screen time among young children: a systematic review. J Obes. 2015;2015: 546925 10.1155/2015/546925 25874123 PMC4383435

[80] De Craemer M, De Decker E, De Bourdeaudhuij I, Vereecken C, Deforche B, Manios Y, et al Correlates of energy balance-related behaviours in preschool children: a systematic review. Obes Rev. 2012;13 Suppl 1: 13–28. 22309062 10.1111/j.1467-789X.2011.00941.x

[81] Duch H, Fisher EM, Ensari I, Harrington A. Screen time use in children under 3 years old: a systematic review of correlates. Int J Behav Nutr Phys Act. 2013;10: 102 10.1186/1479-5868-10-102 23967799 PMC3844496

[82] Carver A, Timperio A, Crawford D. Playing it safe: The influence of neighbourhood safety on children's physical activity—A review. Health & Place. 2008;14: 217–27. 17662638 10.1016/j.healthplace.2007.06.004

[83] Davison KK, Lawson CT. Do attributes in the physical environment influence children's physical activity? A review of the literature. Int J Behav Nutr Phys Act. 2006;3: 1–17. 16872543 10.1186/1479-5868-3-19 PMC1557665

[84] Babic M, Morgan P, Plotnikoff R, Lonsdale C, White R, Lubans D. Physical Activity and Physical Self-Concept in Youth: Systematic Review and Meta-Analysis. Sports Med. 2014;44: 1589–601 13 p. 10.1007/s40279-014-0229-z 25053012

[85] Verloigne M, Van Lippevelde W, Maes L, Brug J, De Bourdeaudhuij I. Family- and school-based correlates of energy balance-related behaviours in 10-12-year-old children: a systematic review within the ENERGY (EuropeaN Energy balance Research to prevent excessive weight Gain among Youth) project. Public Health Nutr. 2012;15: 1380–95 16 p. 10.1017/S1368980011003168 22269173

[86] Boyd TKtgc, Hart Barnett JE, More CM. Evaluating iPad Technology for Enhancing Communication Skills of Children With Autism Spectrum Disorders. Interv Sch Clin. 2015;51: 19–27.

[87] King AM, Thonrieczek M, Voreis G, Scott V. iPad^®^ use in children and young adults with Autism Spectrum Disorder: An observational study. Child Lang Teach Ther. 2014;30: 159–73 15 p.

[88] Aaaet Özen. Effectiveness of Siblings-Delivered iPad Game Activities in Teaching Social Interaction Skills to Children with Autism Spectrum Disorders. Ed Sci: Theory Prac. 2015;15: 1287–303.

[89] Clark MLE, Austin DW, Craike MJ. Professional and Parental Attitudes Toward iPad Application Use in Autism Spectrum Disorder. Focus Autism Other Dev Disabil. 2015;30: 174–81.

[90] American Psychiatric Association. Diagnostic and statistical manual of mental disorders (5th ed) 2013.

[91] May T, Cornish K, Rinehart N. Does gender matter? A one year follow-up of autistic, attention and anxiety symptoms in high-functioning children with autism spectrum disorder. J Autism Dev Disord. 2014;44: 1077–86. 10.1007/s10803-013-1964-y 24105364

[92] King AC, Parkinson KN, Adamson AJ, Murray L, Besson H, Reilly JJ, et al Correlates of objectively measured physical activity and sedentary behaviour in English children. Eur J Public Health. 2010. 10.1093/eurpub/ckq104 20650946

[93] Haug E, Torsheim T, Samdal O. Physical environmental characteristics and individual interests as correlates of physical activity in Norwegian secondary schools: The health behaviour in school-aged children study. Int J Behav Nutr Phys Act. 2008;5: 47 10.1186/1479-5868-5-47 18823545 PMC2564975

[94] van der Horst K, Paw MJ, Twisk JW, van Mechelen W. A brief review on correlates of physical activity and sedentariness in youth. Med Sci Sport Exer. 2007;39: 1241–50. 10.1249/mss.0b013e318059bf35 17762356

[95] Giles-Corti B, Timperio A, Bull F, Pikora T. Understanding physical activity environmental correlates: increased specificity for ecological models. Exerc Sport Sci Rev. 2005;33: 175–81. 16239834 10.1097/00003677-200510000-00005

[96] Cohen KE, Morgan PJ, Plotnikoff RC, Barnett LM, Lubans DR. Improvements in fundamental movement skill competency mediate the effect of the SCORES intervention on physical activity and cardiorespiratory fitness in children. J Sports Sci. 2015;33: 1908–18. 10.1080/02640414.2015.1017734 25716899

[97] Hinkley T, Salmon J, Okely AD, Hesketh K, Crawford D. Correlates of preschool children's physical activity. Am J Prev Med. 2012;43: 159–67. 10.1016/j.amepre.2012.04.020 22813680

[98] Sanders T, Feng X, Fahey PP, Lonsdale C, Astell-Burt T. The influence of neighbourhood green space on children’s physical activity and screen time: Findings from the longitudinal study of Australian children. Int J Behav Nutr Phys Act. 2015;12. 10.1186/s12966-015-0288-z PMC4589082 26419752

[99] McGrath LJ, Hopkins WG, Hinckson EA. Associations of objectively measured built-environment attributes with youth moderate—vigorous physical activity: A systematic review and meta-analysis. Sports Med. 2015;45: 841–65. 10.1007/s40279-015-0301-3 25618013

[100] Viswanathan M, Ansari MT, Berkman ND, Chang S, Hartling L, McPheeters M, et al Assessing the Risk of Bias of Individual Studies in Systematic Reviews of Health Care Interventions. Rockville (MD): Agency for Healthcare Research and Quality, 2012 AHRQ Publication No. 12-EHC047-EF. www.effectivehealthcare.ahrq.gov/. 22479713

[101] Vandenbroucke JP, Von Elm E, Altman DG, Gøtzsche PC, Mulrow CD, Pocock SJ, et al Strengthening the Reporting of Observational Studies in Epidemiology (STROBE): Explanation and Elaboration. PLoS Medicine. 2007;4: e297–1654. 10.1371/journal.pmed.0040297 17941715 PMC2020496

[102] Schulz KF, Altman DG, Moher D. CONSORT 2010 statement: updated guidelines for reporting parallel group randomised trials. BMJ. 2010;340: c332 10.1136/bmj.c332 20332509 PMC2844940

[103] Christakis DA. Rethinking Attention-Deficit/Hyperactivity Disorder. JAMA Pediatr. 2016;170: 109–10. 10.1001/jamapediatrics.2015.3372 26746874

