# Supplementary material for: Physical activity, sedentary behavior and their correlates in children with Autism Spectrum Disorder: A systematic review
Source: PLoS One. 2017 Feb 28;12(2):e0172482. doi: 10.1371/journal.pone.0172482 (PMC5330469; doi:10.1371/journal.pone.0172482)
Supplement: S4 Table — a 1 = strong quality/low risk of bias, 2 = moderate quality/risk of bias, 3 = weak quality/high risk of bias. (DOCX) [file pone.0172482.s004.docx]

S4 Table. Risk of bias^a^ for papers reporting physical activity outcomes

| **Paper** | **Selection Bias** | **Study Design** | **Confounders** | **Data collection methods** | **Withdrawal and dropouts** | **Analyses** | **Global score** |
| --- | --- | --- | --- | --- | --- | --- | --- |
| Ayvazoglu et al 2015^37^ | 3 | 3 | 3 | 3 | 1 | 1 | 3 |
| Boddy et al. 2015^39^ | 2 | 3 | 1 | 1 | 3 | 1 | 3 |
| Bandini et al. 2013^25^ | 2 | 3 | 1 | 3 | 2 | 1 | 3 |
| Dreyer Gillette et al. 2015^35^ | 1 | 3 | 1 | 3 | 3 | 1 | 3 |
| MacDonald et al. 2011 ^33^ | 3 | 3 | 3 | 3 | 1 | 1 | 3 |
| Memari et al. 2012^26^ | 2 | 3 | 1 | 1 | 1 | 1 | 2 |
| Memari et al. 2015^41^ | 2 | 3 | 3 | 1 | 1 | 1 | 3 |
| Must et al. 2015^40^ | 2 | 3 | 1 | 3 | 3 | 1 | 3 |
| Orsmond et al. 2011^28^ | 3 | 3 | 3 | 3 | 1 | 1 | 3 |
| Obrusnikova et al. 2011^27^ | 3 | 2 | 3 | 1 | 2 | 1 | 3 |
| Pan et al, 2005^29^ | 3 | 3 | 3 | 1 | 1 | 1 | 3 |
| Pan et al, 2006^30^ | 2 | 3 | 3 | 2 | 3 | 1 | 3 |
| Pan et al, 2011^31^ | 3 | 3 | 3 | 2 | 1 | 1 | 3 |
| Sandt et al. 2005^32^ | 3 | 3 | 3 | 1 | 3 | 1 | 3 |
| Tatsumi et al., 2015^36^ | 2 | 3 | 3 | 2 | 1 | 1 | 3 |
| Tyler et al. 2014^34^ | 2 | 3 | 3 | 1 | 3 | 1 | 3 |
| Wachnob et al. 2015^38^ | 3 | 3 | 3 | 3 | 1 | 1 | 3 |

^a^ 1= strong quality/low risk of bias, 2 = moderate quality/risk of bias, 3= weak quality/high risk of bias
