# Supplementary material for: Physical activity, sedentary behavior and their correlates in children with Autism Spectrum Disorder: A systematic review
Source: PLoS One. 2017 Feb 28;12(2):e0172482. doi: 10.1371/journal.pone.0172482 (PMC5330469; doi:10.1371/journal.pone.0172482)
Supplement: S5 Table — a 1 = strong quality/low risk of bias, 2 = moderate quality/risk of bias, 3 = weak quality/high risk of bias. (DOCX) [file pone.0172482.s005.docx]

S5 Table. Risk of bias^a^ for papers reporting sedentary behavior outcomes

| **Paper** | **Selection Bias** | **Study Design** | **Confounders** | **Data collection methods** | **Withdrawal and dropouts** | **Analyses** | **Global score** |
| --- | --- | --- | --- | --- | --- | --- | --- |
| Boddy et al. 2015^39^ | 2 | 3 | 1 | 1 | 3 | 1 | 3 |
| Chonchalya et al. 2011^42^ | 2 | 3 | 2 | 3 | 1 | 1 | 3 |
| Dreyer Gillette et al. 2015^35^ | 1 | 3 | 1 | 3 | 3 | 1 | 3 |
| Engelhardt et al. 2013^43^ | 2 | 3 | 1 | 3 | 3 | 1 | 3 |
| Foran et al. 2012^44^ | 3 | 3 | 3 | 3 | 1 | 1 | 3 |
| Kuo et al. 2014^53^ | 2 | 3 | 3 | 3 | 1 | 1 | 3 |
| Kuo et al. 2015^52^ | 2 | 2 | 1 | 3 | 1 | 1 | 2 |
| MacDonald et al. 2011^33^ | 3 | 3 | 3 | 3 | 1 | 1 | 3 |
| MacMullin et al. 2015^51^ | 2 | 3 | 3 | 3 | 1 | 1 | 3 |
| Mazurek et al. 2013^47^ | 2 | 3 | 3 | 3 | 1 | 1 | 3 |
| Mazurek et al. 2013^45^ | 2 | 3 | 1 | 3 | 3 | 1 | 3 |
| Mazurek et al. 2013^46^ | 2 | 3 | 3 | 3 | 1 | 1 | 3 |
| Must et al. 2014^48^ | 2 | 3 | 1 | 2 | 3 | 1 | 3 |
| Must et al. 2015^40^ | 2 | 3 | 1 | 3 | 3 | 1 | 3 |
| Orsmond et al. 2011^28^ | 3 | 2 | 3 | 1 | 2 | 1 | 3 |
| Shane et al. 2008^49^ | 3 | 3 | 3 | 3 | 3 | 1 | 3 |
| Soden et al. 2012^50^ | 2 | 3 | 3 | 3 | 1 | 1 | 3 |
| Tyler et al. 2014^34^ | 2 | 3 | 3 | 1 | 3 | 1 | 3 |

^a^ 1= strong quality/low risk of bias, 2 = moderate quality/risk of bias, 3= weak quality/high risk of bias
